# Pharmacologically Targeting DHODH-Dependent Pyrimidine Metabolism with HSA-Conjugated BRQ Suppresses Leukemogenesis

**DOI:** 10.3390/cancers18193091

**Published:** 2026-09-23

**Authors:** Xiaoxu Zhang, Changhao Tian, Yang Ding, Lei Dong, Jia Yu, Rui Su, Min Gao

**Affiliations:** 1 State Key Laboratory of Common Mechanism Research for Major Diseases, Key Laboratory of RNA and Hematopoietic Regulation, Institute of Basic Medical Sciences and School of Basic Medicine, Chinese Academy of Medical Sciences and Peking Union Medical College, Beijing 100005, China; 2 The Institute of Blood Transfusion, Chinese Academy of Medical Sciences and Peking Union Medical College, Chengdu 610052, China; 3 School of Life Sciences and Health Engineering, Jiangnan University, Wuxi 214122, China; 4 Quantitative Biomedical Research Center, Peter O’Donnell Jr. School of Public Health, UT Southwestern Medical Center, Dallas, TX 7539, USA; 5 Department of Systems Biology and Center for RNA Biology and Therapeutics, Beckman Research Institute of City of Hope, Monrovia, CA 91010, USA; 6 City of Hope Comprehensive Cancer Center, Duarte, CA 91010, USA

**Keywords:** acute myeloid leukemia (AML), PHGDH, metabolism, therapeutic target, ferroptosis, human serum albumin (HSA) conjugation

## Abstract

**Background:** Metabolic reprogramming is a hallmark of acute myeloid leukemia (AML) and provides a rich source of therapeutic vulnerabilities. Among these, *de novo* pyrimidine biosynthesis has emerged as an important metabolic dependency, yet its contribution to AML progression remains incompletely defined. DHODH, a rate-limiting enzyme in this pathway, represents a promising therapeutic target; however, the clinical development of DHODH inhibitors has been constrained by limited efficacy and dose-limiting toxicity. Thus, defining the mechanistic role of DHODH in AML and developing strategies to improve the therapeutic index of DHODH inhibition remain important unmet needs. **Methods:** The Cancer Dependency Map (DepMap) database was utilized to identify AML-essential genes that were preferentially required for AML cell survival. Gene set enrichment analysis (GSEA) was used to characterize metabolic dependencies and identify candidate targets. The functional role of DHODH was subsequently evaluated by using genetic knockout in AML cell lines and patient-derived xenograft (PDX) cells. NSGS mice were used in the “human-in-mouse” xenograft mice model. Metabolic profiling coupled with ^13^C isotope tracing was performed to define DHODH-dependent metabolic alterations, while RNA-seq and genetic rescue assays were used to investigate the mechanisms underlying DHODH-mediated leukemogenesis and ferroptosis. Pharmacologically, we evaluated the DHODH inhibitor BRQ and developed an HSA-based BRQ delivery strategy to improve therapeutic efficacy and reduce systemic toxicity. **Results:** Our study identified DHODH as a critical vulnerability in AML. *DHODH* knockout (KO) significantly suppressed AML cell growth, induced apoptosis in vitro, and markedly inhibited AML progression in vivo, supporting a crucial role of DHODH in leukemogenesis. Metabolic profiling further confirmed the dependence of AML cells on DHODH-mediated *de novo* pyrimidine biosynthesis for leukemic progression. RNA-seq revealed marked activation of the ferroptosis pathway following *DHODH* KO, with *HMOX1* identified as a functionally essential target of DHODH depletion-induced ferroptosis. Pharmacologically, the DHODH inhibitor BRQ phenocopied the antileukemia effects of genetic *DHODH* depletion both in vitro and in vivo. Of note, we have developed an HSA-based delivery strategy aimed at improving therapeutic efficacy while reducing systemic toxicity. Targeting DHODH, particularly with HSA-conjugated BRQ, represents a promising therapeutic strategy for AML.

## Simple Summary

Acute myeloid leukemia (AML) is an aggressive hematopoietic malignancy characterized by myeloid differentiation blockage, clonal expansion of immature progenitor cells, and metabolic dysregulation. Here, we identify *de novo* pyrimidine biosynthesis as a prominent metabolic dependency in AML, with dihydroorotate dehydrogenase (DHODH), a rate-limiting enzyme of this pathway, emerging as a key metabolic vulnerability. Functional studies show that *DHODH* knockout (KO) significantly suppresses cell growth and induces apoptosis in vitro, while substantially delaying leukemia progression in vivo. Mechanistically, DHODH promotes leukemogenesis by sustaining *de novo* pyrimidine metabolism, restraining HMOX1-dependent ferroptosis, and limiting cytosolic accumulation of mitochondrial double-strand RNA (mt-dsRNA). Pharmacologically, we develop a human serum albumin (HSA)-based delivery strategy to improve the therapeutic efficacy and reduce the systemic toxicity of the DHODH inhibitor, brequinar (BRQ). Collectively, our findings establish DHODH as a critical vulnerability in AML and support HSA-conjugated BRQ as a promising therapeutic strategy for DHODH-targeted treatment.

## 1. Introduction

Metabolic reprogramming is a hallmark of cancer [1]. Beyond its well-established roles in supporting cancer cell growth and proliferation, metabolic reprogramming can also reshape the tumor microenvironment and contribute to immune evasion [2]. Pyrimidine nucleotide biosynthesis represents a critical metabolic dependency in rapidly proliferating cancer cells, and pharmacological disruption of this pathway has long been exploited as an anticancer strategy [3]. DHODH, a rate-limiting enzyme in *de novo* pyrimidine metabolism, is localized to the inner mitochondrial membrane, where it couples pyrimidine synthesis to mitochondrial electron transport by transferring electrons to coenzyme Q (CoQ), thereby generating its reduced form, ubiquinol (CoQH_2_) [4,5]. In addition to supporting mitochondrial metabolism, this activity contributes to countering ferroptosis [6]. Accumulating evidence has implicated the critical role of DHODH in cancer initiation and progression, establishing it as a potential therapeutic target across multiple cancer types [7,8,9,10,11,12,13,14,15]. Accordingly, several DHODH inhibitors, including BRQ and BAY2402234, have demonstrated potent antitumor activity in preclinical models [9,11]. However, DHODH inhibitors have shown limited anticancer efficacy in phase 1 clinical trials, and their overall clinical potential remains unclear. Therefore, strategies that enhance therapeutic efficacy while overcoming the limitations of existing DHODH inhibitors are needed to fully exploit DHODH as an anticancer vulnerability.

Acute myeloid leukemia is a fatal form of hematopoietic malignancy [16,17]. More than half of AML patients are either resistant to current chemotherapy or relapse within 1–2 years after achieving initial remission [18]. Consequently, its long-term outcomes remain poor, with only ~30% of AML patients surviving beyond 5 years [19]. These clinical challenges underscore the urgent need to identify new therapeutic vulnerabilities, particularly those that support leukemia persistence and treatment resistance.

In contrast to normal hematopoietic stem cells (HSCs), which predominantly depend on glycolysis, AML cells—especially leukemia stem cells (LSCs)—display a heightened dependence on mitochondrial oxidative phosphorylation (OxPhos) for energy production and survival [20,21]. Such metabolic reprogramming is increasingly recognized as a key feature of hematological malignancies and has provided a foundation for the development of metabolism-directed therapies. Indeed, preclinical and clinical studies have demonstrated promising therapeutic benefits from targeting mitochondrion-associated vulnerabilities in AML, such as BCL2- and IDH2-directed therapies [22,23,24,25]. However, the contribution of *de novo* pyrimidine metabolism, particularly DHODH, to AML maintenance and progression remains incompletely understood. Moreover, whether pharmacologically targeting DHODH can provide an effective and therapeutically tractable antileukemic strategy remains to be fully established.

Here, we identify *de novo* pyrimidine biosynthesis as a prominent metabolic dependency in AML and establish DHODH as a critical driver of AML cell survival and leukemogenesis. We further demonstrate that pharmacological inhibition of DHODH with BRQ exerts potent antileukemic effects. Importantly, we develop HSA-conjugated BRQ nanoparticles that preserve promising antileukemic activity while markedly reducing systemic toxicity, thereby conferring a dual therapeutic advantage through potent DHODH inhibition and improved treatment tolerability in AML.

## 2. Materials and Methods

### 2.1. Cell Culture

The human leukemia cells, MOLM-13(RRID: CVCL_2119; DSMZ [ACC-554]) and MV4-11(RRID: CVCL_0064; DSMZ [ACC-102]) were cultured in RPMI1640 (11875119, Gibco) supplemented with 10% FBS; Mono-mac-6 (RRID: 340 CVCL_1426; DSMZ [ACC-124]) was maintained in RPMI1640 supplemented with 10% FBS, 1 × non-essential amino acids (11140050, Thermo Fisher Scientific, Waltham, MA, USA), 1 mM sodium pyruvate (113600 70, Thermo Fisher Scientific, Waltham, MA, USA), and 10 mg/mL human insulin (12585014, Thermo Fisher Scientific, Waltham, MA, USA). The AML PDX-148 cells were cultured in IMDM (12440061, Gibco, New York, Waltham, MA, USA) supplemented with 20% FBS, 10 ng/mL of human cytokines SCF (250-03, PeproTech, Cranbury, NJ, USA), TPO (300-18, PeproTechFLT3 (300-19, PeproTech), IL-3 (213-13, PeproTech), and IL-6 (200-06, PeproTech); HEK293T (RRID: CVCL_0063; ATCC [CRL-3216]) was maintained in DMEM supplemented with 10% FBS. All cells were maintained at 37 °C in a humidified atmosphere with 5% CO2. All cell lines were tested and confirmed to be mycoplasma-free before proceeding with further studies.

### 2.2. Animals

The “human-in-mouse” xenograft models were established using 6–8-week-old NSGS mice (NOD.Cg-Prkdc*^scid^* Il2rg*^tm1Wjl^* Tg (CMV-IL3, CSF2, KITLG)1Eav/MLoySzJ; RRID: IMSR_JAX:013062). The mice were purchased from the Jackson Laboratory and bred under pathogen-free conditions. All mice were maintained on a 12 h light/12 h dark cycle with food and water provided ad libitum and randomly assigned to experimental groups. All animal experiments were performed in accordance with institutional guidelines and approved by the Institutional Animal Care and Use Committee (IACUC) at City of Hope.

### 2.3. Lentivirus Production and Infection

All lentiviruses used in this study were based on the second-generation packaging system. Briefly, 0.75 µg of psPAX2 (12260, Addgene, Watertown, MA, USA), 0.25 µg of pMD2.G (12259, Addgene), plus 1 µg of target plasmid were co-transfected into HEK293T cells using X-tremeGENE HP DNA Transfection Reagent (6366546001, Roche; 3 µL for one well in a 6-well plate), following medium replacement after 4–6 h. Virus supernatants were collected at 48 and 72 h post-transfection, centrifuged at 3000× *g* for 15 min at 4 °C to remove cellular debris, and stored at 4 °C for short-term or −80 °C for long-term storage. For human AML cells, the viral supernatant was added to cells along with 4 µg/mL polybrene (H9268, Sigma-Aldrich, St. Louis, MO, USA), followed by two rounds of spinoculation at 1200 rpm and 32 °C for 2 h. For murine cells, the viral supernatant was added to cells along with 4 µg/mL polybrene (H9268, Sigma-Aldrichfollowed by two rounds of spinoculation at 2000 rpm and 32 °C for 3 h. After 24 h, the medium was replaced with fresh medium, and the lentivirus was removed. Infected cells were selected with the following antibiotics starting at 48 h post-infection: 200 µg/mL hygromycin B (ant-hg-1, 420 Invivogen, San Diego, CA, USA) for sgRNAs, 1.5 µg/mL puromycin (ant-pr-1, Invivogen) for pLKO.1. The knockout efficiency was validated by RT-qPCR.

### 2.4. RNA Extraction and Real-Time Quantitative (RT-PCR) Analysis

Total RNA was extracted using QIAzol Lysis Reagent (79306, QIAGEN, Germantown, MD, USA) according to the manufacturer’s instructions. Reverse transcription was performed with 500–1000 ng of total RNA using the QuantiTect Reverse Transcription kit (205314, QIAGEN) according to the manufacturer’s instructions. qPCR was performed using SYBR Green Master Mix (A25778, Thermo Fisher Scientific) on a QuantStudio^TM^ 7 Flex Real-Time PCR system (Applied Biosystems, Foster City, CA, USA). GAPDH, 18S rRNA, or 28S rRNA were used as endogenous controls, and all reactions were performed in triplicate.

### 2.5. Immunofluorescence Staining

Cells were washed three times with PBS and subsequently fixed in 4% paraformaldehyde for 15 min at room temperature, followed by three washes with PBS. Cells were incubated with 1 × permeabilization buffer (00-8333-56, Thermo Fisher) for 15 min at room temperature and washed three times with PBS. The slices were then mounted with In Situ Mounting Medium DAPI (DUO82040, Sigma-Aldrich). Images were acquired using a Zeiss LSM 880 confocal microscope (Zeiss, Germany), and colocalization was analyzed using ZEN 3.1 (blue edition).

### 2.6. Cell Viability and Proliferation Assay

Cell growth and proliferation were tested by the TACS MTT Cell Proliferation Assay (4890-050-K, R&D Systems, Minneapolis, MN, USA). A total of 7000–10,000 live cells in a total volume of 100 µL were seeded into 96-well plates. Following the manufacturer’s instructions, 10 µL of dye solution was added to each well at defined time points, followed by incubation at 37 °C in a 5% CO_2_ humidified incubator for 4 h. Then, 100 µL of detergent reagent was added to each well to quench the reaction. Absorbance was measured at 570 nm using the BioTek Gen5 system (BioTek, Winooski, VT, USA) the next day.

### 2.7. Flow Cytometry Analysis

(1) To assess ferroptosis, approximately 0.5 × 10^6^ cells were collected, resuspended in 500 µL of medium, and stained with 10 µM BODIPY (121207-31-6, MCE, NJ, USA) for 30 min at room temperature. Samples were then immediately analyzed by flow cytometry.

(2) To assess cell apoptosis, the PE Annexin V Apoptosis Detection Kit I (559763, BD Biosciences, San Jose, CA, USA) was utilized following the manufacturer’s instructions. Briefly, 0.5 × 10^6^ cells were collected, washed twice with cold PBS, and resuspended in 100 µL of 1× Binding Buffer. A total of 5 µL of PE Annexin V and 5 µL of 7-AAD were added to the cells and mixed well, followed by incubation at room temperature in the dark for 15 min. The samples were subsequently treated with 400 µL of 1× Binding Buffer and immediately analyzed by flow cytometry.

(3) To assess the leukemia burden of recipient mice, mononuclear cells were collected from peripheral blood, bone marrow, spleen, and liver at the defined time points from each group. The cells were treated with Ammonium Chloride Solution (07850, STEMCELL Technologies, Vancouver, BC, Canada) at room temperature for 15 min, following centrifugation at 1800 rpm for 8 min. Cells were stained with APC-human CD45 antibody (17-9459-42, eBioscience, San Diego, CA, USA) in FACS buffer at 4 °C for 30 min, then washed with PBS and analyzed by flow cytometry. All flow cytometry was performed using a BD Fortessa X20, and data were analyzed with FlowJo v10.0 software.

### 2.8. Preparation and Characterization of HSA-Based Nanoparticles

HSA-BRQ nanoparticles were prepared by self-assembly of HSA and BRQ, with HSA serving as the carrier and BRQ encapsulated within the albumin matrix. HSA protein (12.5 mL, 2.5 mg/mL) was dissolved in PBS solution and adjusted to pH 5.0 using hydrochloric acid. Brequinar (6.0 mg) was dissolved in 3 mL of DMSO and slowly added to the HSA protein solution, followed by stirring overnight at 4 °C. The reaction mixture was subsequently neutralized with sodium hydroxide and allowed to react for an additional 4 h. The resulting mixture was centrifuged at 6000 rpm for 1 min to remove insoluble brequinar. The supernatant was then subjected to three rounds of ultrafiltration against PBS using centrifugal filter units with a molecular-weight cutoff (MWCO) of 10 kDa to remove DMSO. The resulting nanoparticles were frozen at −80 °C overnight and subsequently lyophilized. The hydrodynamic diameter and zeta potential of the protein nanoparticles were measured using a Malvern Zetasizer Nano ZS. Prior to measurement, all samples were diluted with deionized water to a final concentration of 0.5 mg/mL. The prepared nanoparticles were diluted in 80% DMSO to disrupt the nanoparticle structure and release the encapsulated drug, then quantified using a UV–visible spectrophotometer, and the drug-loading capacity was calculated accordingly. The in vitro release of BRQ from HSA-BRQ nanoparticles was carried out at 37 °C in a dialysis bag (MWCO = 35 KDa) under continuous stirring. PBS (pH 7.4) was used as the release medium. At predetermined time points, 1 mL of the release medium was withdrawn and analyzed using a UV–visible spectrophotometer to quantify the released BRQ. The release profile was expressed as the BRQ concentration versus time. The release profile of free BRQ from the dialysis bag was also determined as a control. To assess stability in serum, HSA-BRQ nanoparticles were dispersed in PBS containing 10% (*v*/*v*) fetal bovine serum (FBS) at 37 °C, and their hydrodynamic size was measured by dynamic light scattering (DLS) after 24 and 48 h.

### 2.9. Bioluminescence Imaging

In vivo bioluminescence imaging was conducted to monitor the leukemia burden in NSGS recipient mice. Mice were weighed and intraperitoneally injected with 150 mg/kg D-luciferin (LUCK-2G, GoldBio, St. Louis, MO, USA), followed by anesthesia with isoflurane. After 10 min, whole-body bioluminescence imaging was performed using Lago X (Spectral Instruments Imaging). Signals were quantified as radiance (photons/s/cm^2^/sr), and pseudocolor images indicated signal intensity corresponding to leukemia burden.

### 2.10. In Vivo Efficacy and Organ Toxicity

A total of 1 × 10^6^ MOLM-13 cells in 100 μL of PBS were injected into NSGS mice via the tail vein. Seven days after inoculation, mice were randomly assigned to three groups (n = 5 per group): PBS control, free BRQ, and HSA-BRQ. Free BRQ and HSA-BRQ were administered via tail vein injection at an equivalent BRQ dose of 10 mg/kg body weight every 2 days for 2 consecutive weeks. The control group received an equal volume of PBS on the same schedule. For survival analysis, a separate cohort of mice (n = 5 per group) was used. Mice were monitored daily from the day of tumor inoculation. Moribund mice or mice with more than 20% body weight loss were euthanized according to IACUC guidelines, and this was recorded as the survival endpoint. Kaplan–Meier survival curves were plotted and compared using the log-rank test. For endpoint analyses, another cohort of mice (n = 5 per group) was used. Blood samples were collected via orbital sinus or cardiac puncture into EDTA-coated tubes at the end of treatment. Complete blood count (CBC) was measured using an automated hematology analyzer. Subsequently, mice were sacrificed, and spleens were removed and weighed. Regarding the toxicity study, normal NSGS mice without MOLM-13 engraftment were randomly divided into PBS, free BRQ, and HSA-BRQ groups (n = 5 per group) and received the same dosing regimen as described above. Body weight was recorded every 2 days. At day 28, after the first administration, mice were sacrificed. The heart, liver, spleen, lung, and kidney were harvested, fixed in 4% paraformaldehyde, embedded in paraffin, sectioned, and stained with hematoxylin and eosin (H&E). Histological changes were examined under a light microscope.

### 2.11. RNA-Sequencing and Data Analysis

Total RNA was extracted from Mono-Mac-Cas9 cells (sgNS, sgDHODH-1; two biological replicates for each group) using QIAzol Lysis Reagent according to the manufacturer’s instructions. RNA concentration was determined using NanoDrop 2000 (Thermo Fisher Scientific), and RNA integrity was assessed with a 2100 Bioanalyzer (Agilent Technologies). Poly(A)+ RNA was enriched from total RNA, and sequencing libraries were prepared using the KAPA Stranded mRNA-Seq Kit for Illumina platforms (Kapa Biosystems, Wilmington, MA, USA) with 10 cycles of PCR amplification. Libraries were purified using the AxyPrep Mag PCR Clean-up Kit (Thermo Fisher Scientific) and sequenced on an Illumina NovaSeq platform to generate 100 bp paired-end reads (PE100).

After sequencing, reads were trimmed and mapped to the human reference genome (GRCh38) using STAR. Transcript abundances were quantified by RSEM and normalized by ERCC. Differentially expressed genes were subjected to Gene Set Enrichment Analysis (GSEA) to identify enriched pathways. RNA-seq genome browser tracks were visualized using IGV.

### 2.12. Metabolite Extraction and Mass Spectrometry-Based Metabolomics Analysis

Metabolites were extracted from Mono-Mac-6-Cas9 cells (sgNS, sgDHODH-1; three biological replicates for each group). A total of 1.5 × 10^6^ AML cells were seeded in a 6-well plate and cultured overnight in 3 mL of medium containing 10 mM ^13^C_6_-glucose (CLM-1396-1, Cambridge Isotope Laboratories, Tewksbury, MA, USA) and dialyzed FBS (SH3007902, Thermo Fisher Scientific) in place of normal glucose and FBS. Cells were washed once with ice-cold 5% mannitol and resuspended in 1 mL of 80% methanol (pre-cooled at −80 °C) with 1 nmol of norvaline (N7502, Sigma-Aldrich). Cell suspensions were incubated at −80 °C for 1 h, thawed on ice, vortexed for 20 s, and centrifuged at 16,000× *g* for 15 min at 4 °C to collect supernatants. The supernatants were then dried using a Vacufuge Plus (30 °C, Eppendorf, Hamburg, Germany ) and stored at −80 °C. The dried metabolites were subjected to mass spectrometry-based metabolite profiling at the UCLA Metabolomics Center. Metabolic pathway impact analyses were performed using MetaboAnalyst (https://www.metaboanalyst.ca/MetaboAnalyst/, accessed on 5 January 2026).

### 2.13. Quantification and Statistical Analysis

Statistical significance was assessed by either unpaired Student’s *t*-tests, one-way ANOVA, or two-way ANOVA. Pearson correlation analysis was performed using Pearson’s test, and survival curves were compared using the log-rank test. Statistical significance was defined as *p* < 0.05 (noted as *), *p* < 0.01 (**), or *p* < 0.001 (***). RNA-seq was performed with two biological replicates, and mass spectrometry-based metabolomics analysis was performed with three biological replicates. All statistical analyses were visualized using GraphPad Prism 10 (GraphPad Software). All RT-qPCRs shown are representative of at least three independent experiments. The number of independent experiments or mice per group is indicated in the corresponding figure legends.

## 3. Results

### 3.1. De Novo Pyrimidine Synthesis Is Required for AML

To identify potential therapeutic vulnerabilities in AML, we performed an unbiased analysis using data from the Cancer Dependency Map (DepMap) project. We leveraged genome-scale CRISPR-Cas9 dependency data from more than 1200 cancer cell lines to systematically identify genes preferentially required for AML cell survival [26]. By analyzing CRISPR dependency scores, we classified genes as either AML-essential genes or AML non-essential genes according to their dependency in AML cell lines relative to other cancer cell lines. This analysis identified 568 AML essential genes, which exhibited significantly lower CRISPR scores in AML cell lines than in other cancer cell lines (Figure 1A).

Gene set enrichment analysis (GSEA) of these AML-essential genes revealed pronounced enrichment of metabolism-related pathways (Figure 1B). Notably, “biosynthesis of cofactors” emerged as the most significantly enriched pathway (Figure 1B). Among the core-enriched genes contributing to this pathway, 11 genes showed significantly higher expression levels in AML patients than in healthy controls (Figure 1C). Further pathway analysis of these core-enriched genes revealed a substantial enrichment of pyrimidine metabolism, highlighting *DHODH*, *CTPS1*, and *CAD* as key components of this pathway (Figure 1D).

Pyrimidine nucleotide biosynthesis is a critical metabolic pathway that supports the rapid proliferation of cancer cells, and pharmacological disruption of this pathway has long been exploited as an anticancer strategy [3]. Among the enzymes involved in pyrimidine biosynthesis, DHODH, a rate-limiting enzyme in *de novo* pyrimidine biosynthesis, catalyzes the oxidation of dihydroorotate to orotate and thereby plays a central role in sustaining intracellular pyrimidine pools [27] (Figure 1E). Notably, among the pyrimidine biosynthesis-related genes examined, *DHODH* exhibited the most robust upregulation in AML patients compared with healthy controls (Figure 1F).

Collectively, these findings suggest that *de novo* pyrimidine biosynthesis is a critical metabolic dependency in AML and identifies DHODH as a potential vulnerability.

### 3.2. DHODH Promotes Leukemogenesis

We next investigated the specific functional significance of DHODH in AML. *DHODH* KO significantly suppressed AML cell proliferation (Figure 2A), drove monocyte-to-macrophage differentiation (Figure 2B), and induced apoptosis (Figure 2C) in both AML cell lines and primary patient-derived xenograft (PDX) cells. These findings demonstrate that DHODH is required to sustain AML cell growth and survival in vitro.

To confirm the role of DHODH in leukemogenesis in vivo, we employed an AML “human-in-mouse” xenograft model. Loss of *DHODH* significantly prolonged mouse survival (Figure 2D), while in vivo bioluminescence imaging demonstrated a substantial reduction in leukemia burden following *DHODH* KO (Figure 2E, F). Consistently, *DHODH* depletion profoundly impaired leukemic engraftment (Figure 2G) and alleviated splenomegaly (Figure 2H) at the disease endpoint. H&E staining further revealed that *DHODH* KO remarkably suppressed leukemic infiltration into the liver compared with the control group (Figure 2I). These results independently validate the role of DHODH in promoting AML progression.

Taken together, these findings establish DHODH as a critical driver of leukemogenesis and support its potential as a therapeutic vulnerability in AML.

### 3.3. Pyrimidine-Synthesis-Dependent Activity of DHODH in AML

Given our findings that DHODH, a well-known rate-limiting enzyme in *de novo* pyrimidine biosynthesis, promoted leukemogenesis, together with increasing evidence that metabolic rewiring is a hallmark of hematological malignancies, we next sought to define the metabolic plasticity of DHODH in AML. To systemically evaluate the role of DHODH in metabolism, we performed stable isotope (^13^C) tracing coupled with liquid chromatography–mass spectrometry (LC-MS) in AML cells following *DHODH* KO (Table S1). Unbiased metabolomic profiling showed robust and reproducible metabolic alterations upon *DHODH* depletion (Figure 3A). Further analysis identified hundreds of metabolites whose abundances were significantly dysregulated following *DHODH* KO. Strikingly, dihydroorotate (DHO), the direct substrate of DHODH, was the most markedly accumulated metabolite following *DHODH* KO (Figure 3B).

Metabolite set enrichment analysis (MSEA) of significantly increased metabolites revealed prominent enrichments of (i) glutathione metabolism, (ii) one-carbon pool by folate, and (iii) purine metabolism (Figure 3C). In contrast, pathway analysis of significantly decreased metabolites identified marked enrichments of (i) pyrimidine metabolism, (ii) phenylalanine, tyrosine, and tryptophan biosynthesis, and (iii) glycerophospholipid metabolism (Figure 3D). Consistent with the canonical function of DHODH, pyrimidine metabolism emerged as the most significantly decreased metabolic pathway upon *DHODH* KO, with pronounced reductions in downstream pyrimidine intermediates, such as Uridine, UTP, and CTP (Figure 3E–G).

These findings demonstrate that DHODH is critically required to sustain *de novo* pyrimidine biosynthesis in AML and its depletion induces broad metabolic remodeling.

### 3.4. DHODH Loss Induces Ferroptosis Through HMOX1

To further delineate the molecular mechanisms by which *DHODH* promotes AML cell survival and leukemogenesis, we conducted transcriptome-wide RNA-sequencing (RNA-seq) in AML cells following *DHODH* KO (Figure 4A and Table S2). RNA-seq identified 508 significantly downregulated transcripts (*p* < 0.05; log_2_[fold change] < −0.3) and 781 significantly upregulated transcripts (*p* < 0.05; log_2_[fold change] > 0.3) (Figure 4B). Of note, pathway enrichment analysis revealed predominant activation of metabolism- and immune-related pathways upon *DHODH* KO, with the ferroptosis pathway emerging as the most substantially affected pathway (Figure 4C). Consistent with these findings, a flow cytometric assay demonstrated that *DHODH* depletion markedly induced ferroptotic cell death (Figure 4D).

Since mitochondria harbor their own genome and constitutively generate mitochondrial double-strand RNA (mt-dsRNA), whose cytosolic accumulation intriguingly induces ferroptosis [28], we next examined whether *DHODH* depletion affects mt-dsRNA localization. Immunofluorescence (IF) analysis revealed a pronounced redistribution of mt-dsRNA from mitochondria to the cytoplasm in AML cells following *DHODH* KO (Figure 4E), suggesting that mt-dsRNA release may contribute to ferroptosis induced by *DHODH* depletion.

To determine the functionally essential downstream target of DHODH-mediated ferroptosis in AML, we focused on the top core-enriched genes within the ferroptosis pathway. Among these, *heme oxygenase-1* (*HMOX1)* exhibited the most robust upregulation following *DHODH* KO (Figure 4F). HMOX1 plays a central role in iron homeostasis by catalyzing the degradation of heme into biliverdin, carbon monoxide, and ferrous iron; excessive HMOX1 activity can promote iron accumulation, mitochondrial dysfunction, and ferroptosis [28,29]. Moreover, *HMOX1* expression was significantly negatively correlated with *DHODH* expression in AML (Figure 4G). Additional RT-qPCR analyses confirmed that *DHODH* KO markedly increased *HMOX1* expression (Figure 4H). We next performed genetic rescue assays to determine whether *HMOX1* functions as a bona fide downstream target of DHODH in AML. Compared with *DHODH* KO, the depletion of *HMOX1* alone exerted a relatively modest effect on AML cell growth inhibition and apoptosis (Figure 4I,J). Importantly, the growth inhibition and apoptosis induced by *DHODH* KO could be partially reversed by *HMOX1* KO (Figure 4I,J). More strikingly, *HMOX1* KO substantially attenuated *DHODH* KO-induced ferroptosis (Figure 4K), indicating that DHODH promotes leukemogenesis, at least in part, by restraining HMOX1-dependent ferroptosis. Collectively, these findings identify HMOX1 as a critical downstream mediator of DHODH depletion-induced ferroptosis in AML.

### 3.5. HSA-Based BRQ Delivery Improves Efficacy and Tolerability in AML

Given that DHODH is a validated vulnerability in AML, we then evaluated whether pharmacologically targeting DHODH with its inhibitor, BRQ (DUP 785; NSC 368390), could recapitulate the antileukemic effects of genetic *DHODH* depletion. We found that BRQ treatment effectively reduced the viability of multiple AML cell lines and PDX cells (Figure 5A). Moreover, BRQ treatment significantly decreased *DHODH* expression (Figure 5B). Consistent with the effect of *DHODH* loss, BRQ treatment notably increased *HMOX1* expression and induced ferroptosis (Figure 5B,C). Moreover, the exogenous Uridine rescue assay in AML cells showed that Uridine substantially rescued *DHODH* inhibition-induced ferroptosis, decreased *HMOX1* mRNA expression, and suppressed mt-dsRNA cytosolic leakage (Figure S1).

Although BRQ has demonstrated promising antileukemic activity, its clinical development has been constrained by dose-limiting toxicity [30,31]. To overcome this limitation, we developed an HSA-based delivery strategy to determine whether pharmacological inhibition of DHODH could be achieved with improved therapeutic efficacy and reduced systemic toxicity (Figure 5D). HSA-BRQ nanoparticles were prepared by self-assembly of HSA and BRQ. The resulting nanoparticles had an average hydrodynamic diameter of 153 ± 9.3 nm and a PDI of 0.233 ± 0.012 (Figure 5E), indicating a relatively narrow size distribution. The drug-loading capacity was 1.45 ± 0.42%. The zeta potential of HSA-BRQ nanoparticles was −11.7 ± 1.08 mV. This slightly negative surface charge, together with the albumin shell, may reduce non-specific interactions and contribute to the colloidal stability of the nanoparticles. The serum stability assay showed that the hydrodynamic size of HSA-BRQ nanoparticles remained essentially unchanged over 48 h in PBS containing 10% FBS (Figure S2). In addition, the in vitro release profile showed that BRQ was released from HSA-BRQ nanoparticles in a sustained manner, whereas free BRQ was rapidly released, confirming that the nanoparticles prevented premature drug release and maintained BRQ stability during circulation (Figure S3). These results indicate that HSA-BRQ nanoparticles possess suitable physicochemical properties for further in vivo evaluation. Notably, HSA-conjugated BRQ retained potent antileukemic activity, significantly suppressing AML cell proliferation with IC_50_ values in the nanomolar range (Figure 5F). Immunofluorescence analysis further demonstrated efficient intracellular delivery of HSA-conjugated BRQ into AML cells, providing a basis for subsequent evaluation of its in vivo anti-AML efficacy and toxicity (Figure 5G).

We next evaluated the in vivo antileukemic efficacy and tolerability of BRQ and HSA-conjugated BRQ. As shown in Figure 5H, BRQ alone exhibited moderate antileukemic activity. Notably, compared with BRQ alone, HSA-conjugated BRQ more effectively inhibited AML initiation and significantly prolonged overall survival. Consistent with its improved therapeutic efficacy, HSA-conjugated BRQ also displayed a markedly improved safety profile. BRQ treatment caused significant body weight loss, whereas mice treated with HSA-conjugated BRQ showed no significant difference in body weight compared with control mice (Figure 5I). H&E staining revealed that BRQ treatment induced pronounced tissue injury in multiple organs, including heart, liver, spleen, lung, and kidney (Figure 5J). In contrast, HSA-conjugated BRQ treatment dramatically attenuated these pathological abnormalities (Figure 5J). Moreover, treatments of both BRQ and HSA-conjugated BRQ substantially reduced splenomegaly (Figure 5K). An analysis of peripheral blood further demonstrated that HSA-conjugated BRQ substantially alleviated the BRQ-induced reductions in populations of white blood cells (WBC) and platelets (PLT) (Figure 5L).

Collectively, these findings indicate that HSA-based delivery preserves the antileukemic activity of BRQ while substantially reducing its systemic toxicity.

## 4. Discussion

Our current study establishes *de novo* pyrimidine biosynthesis as a prominent metabolic dependency in AML and identifies DHODH as a key metabolic vulnerability that promotes AML progression by sustaining *de novo* pyrimidine biosynthesis and suppressing HMOX1-dependent ferroptosis. We demonstrate that both pharmacological and genetic inhibition of DHODH exert potent antileukemia effects in both AML cell lines and PDX cells and substantially reduce leukemia burden and prolong AML mice survival in vivo. Mechanistically, we identify *HMOX1* as a functionally essential target of *DHODH* depletion-induced ferroptosis. Pharmacologically targeting DHODH with BRQ recapitulates the antileukemic effects of genetic *DHODH* depletion, whereas HSA-based delivery further enhances its therapeutic efficacy while mitigating systemic toxicity. Collectively, these findings establish a mechanistic link between *de novo* pyrimidine metabolism and ferroptosis regulation in AML and provide a therapeutic strategy to improve the efficacy and tolerability of DHODH-targeted therapy.

Pyrimidine nucleotides are critical for RNA and DNA synthesis and therefore play a key role in tumor cell proliferation [32]. DHODH, a key enzyme in pyrimidine biosynthesis, contributes to pyrimidine metabolic reprogramming and tumorigenesis across numerous preclinical cancer models [8,10,11,13,14,15]. Previous studies have demonstrated that *DHODH* inhibition depletes intracellular pyrimidine pools, promotes myeloid differentiation, and suppresses leukemia progression in multiple AML models [9,33,34]. Through an unbiased genome-scale dependency analysis, we independently identified *de novo* pyrimidine metabolism as a preferential dependency of AML cells, with its rate-limiting enzyme, DHODH, emerging as a particularly prominent vulnerability.

Although the role of DHODH in nucleotide metabolism and cell proliferation is well established, its connection to ferroptosis has only begun to emerge recently, and whether DHODH serves as a major contributor to ferroptosis susceptibility in AML remains incompletely understood [34,35,36]. Mechanistically, DHODH transfers electrons to mitochondrial ubiquinone, generating ubiquinol, a radical-trapping antioxidant that protects mitochondrial membranes from lipid peroxidation. *DHODH* inactivation has been shown to impair ubiquinone reduction, thereby promoting lipid peroxidation and ferropotic cell death, particularly in cancer cells with low GPX4 expression [35]. Subsequent studies further confirmed the effects of *DHODH* depletion in inducing ferroptosis through disruption of phospholipid metabolism, including CDP-choline abundance in solid tumors [6]. Notably, our findings extend these observations to AML by identifying *HMOX1* as a bona fide downstream target of *DHODH*-depletion-induced ferroptosis. *DHODH* loss markedly upregulated *HMOX1* expression and was accompanied by a profound increase in ferroptosis. Genetic rescue experiments further demonstrated that *HMOX1* KO substantially attenuated *DHODH*-KO-induced ferroptosis. These findings extend previous studies by implicating *HMOX1* as an important component of the ferroptosis response following *DHODH* KO in AML.

Several DHODH inhibitors have been developed, including BAY2402234, JNJ-74856665, AG-636, and BRQ. Among these, BRQ has been evaluated in multiple phase I clinical trials across several cancer types, including AML [37,38,39,40,41,42,43]. However, its clinical development has been limited due to the narrow therapeutic window, dose-dependent toxicity, and insufficient clinical efficacy [39,40]. In addition, off-target effects have been reported for several DHODH inhibitors [44,45]. These limitations prompt us to develop an HSA-based BRQ delivery strategy. HSA-based nanoparticles have emerged as a promising drug-delivery platform because of their bioavailability and potential to improve drug solubility, pharmacokinetics, and tissue distribution while reducing systemic toxicity [46,47]. In our study, HSA-conjugated BRQ nanoparticles exhibited enhanced antileukemic efficacy while substantially reducing systemic toxicity compared with BRQ alone, highlighting HSA-based delivery as a potential strategy to improve the therapeutic index of DHODH inhibition in AML.

Emerging evidence suggests that DHODH may also function as an important metabolic regulator of tumor immune evasion and the tumor microenvironment. *DHODH* depletion has been shown to enhance CD8^+^ T-cell-mediated killing and increase IFNγ production by CD8^+^ T cells in association with ferroptotic cell death [6]. Genetic depletion or pharmacological inhibition of *DHODH* also enhanced cancer cell sensitivity to anti-PD-1 therapy [6]. Moreover, BRQ was found to inhibit the generation of myeloid-derived suppressor cells, a major immunosuppressive cell population within the tumor microenvironment, from early-stage myeloid progenitors [48]. These observations raise the intriguing possibility that DHODH targeting may exert antitumor effects not only through leukemia-intrinsic metabolic and ferroptosis mechanisms but also through modulation of the immune microenvironment. Whether and how DHODH influences antitumor immunity or T-cell responses in AML therefore warrants further investigation.

## 5. Conclusions

Collectively, we identify *de novo* pyrimidine biosynthesis as a prominent metabolic dependency in AML and establish DHODH as a critical driver of leukemogenesis. Importantly, we develop an HSA-conjugated BRQ formulation that preserves promising antileukemic activity while markedly reducing systemic toxicity, thereby conferring a dual therapeutic advantage through potent DHODH inhibition and improved treatment tolerability in AML.

## Figures and Tables

**Figure 1:**
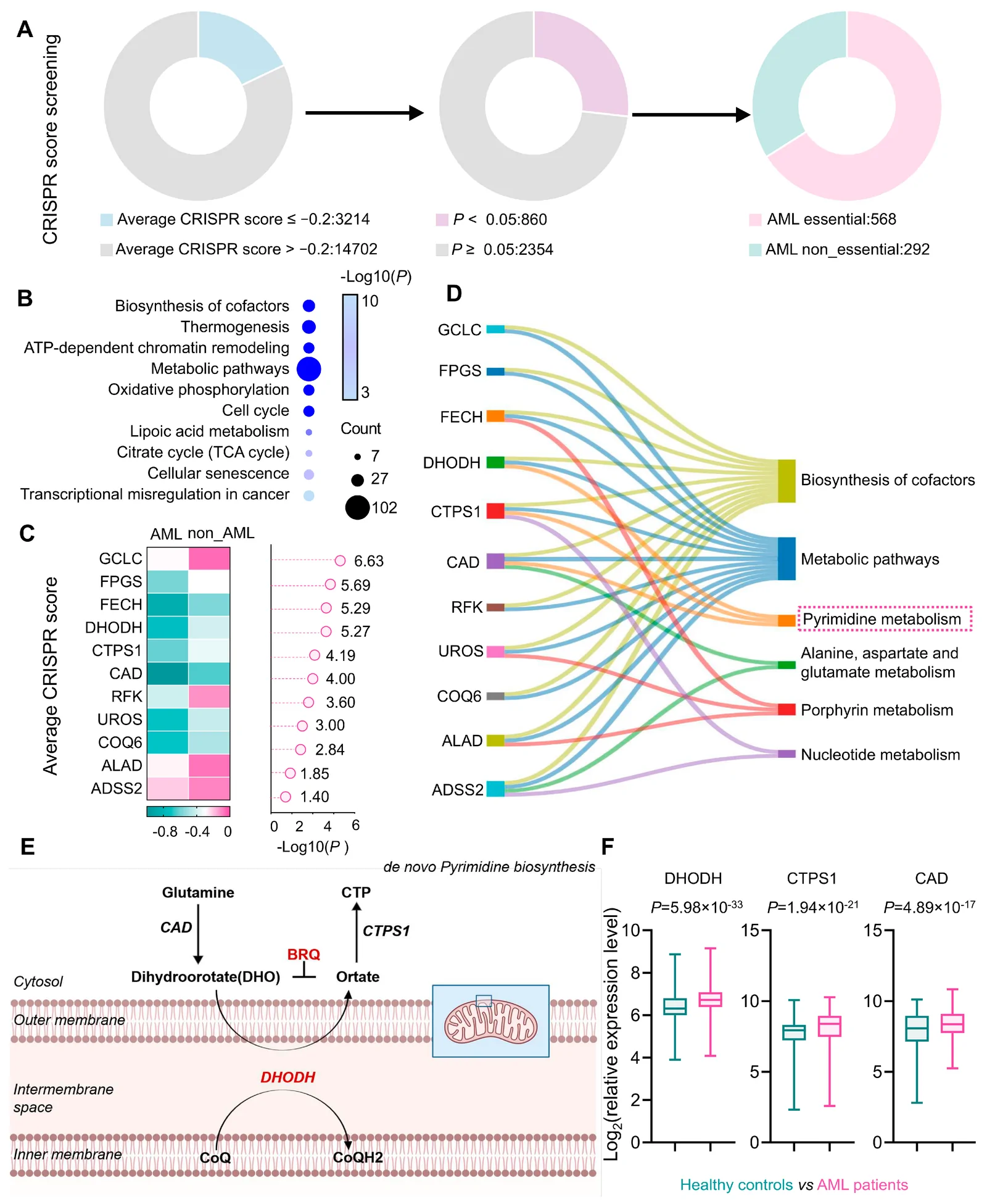
*De novo* pyrimidine biosynthesis is a critical metabolic dependency in AML, and DHODH is a potential vulnerability. (**A**) Analysis workflow used to identify the AML-essential genes for AML cell survival: (**left**) according to the average CRISPR scores across 1200 cell lines, 3214 genes show an average CRISPR score ≤ −0.2, whereas 14,702 genes show an average CRISPR score > −0.2; (**middle**) 860 genes show a significant (*p* < 0.05) difference in CRISPR scores in AML cell lines compared with other cancer cell lines, whereas 2354 genes show no significant difference (*p* ≥ 0.05); (**right**) 568 genes are identified as AML-essential genes, which show significantly lower CRISPR scores in AML cell lines than in other cancer cell lines, whereas 292 genes are identified as AML-non-essential genes. Data from DepMap (24Q4 version) are utilized for analysis. (**B**) Pathway analysis showing the top 10 core-enriched pathways of 568 AML-essential genes identified in Figure 1A. (**C**) Average CRISPR scores of 11 genes that are enriched in the ‘biosynthesis of cofactors’ pathway and show significantly elevated expression in AML patients compared with healthy controls. (**D**) Pathway analysis of 11 genes, shown in Figure 1C, identifying 6 core-enriched pathways. *de novo* pyrimidine pathway is highlighted with pick box. (**E**) Schematic of the *de novo* pyrimidine pathway. (**F**) Box plots depicting transcript levels of *DHODH*, *CTPS1*, and *CAD* in healthy controls and AML patients.

**Figure 2:**
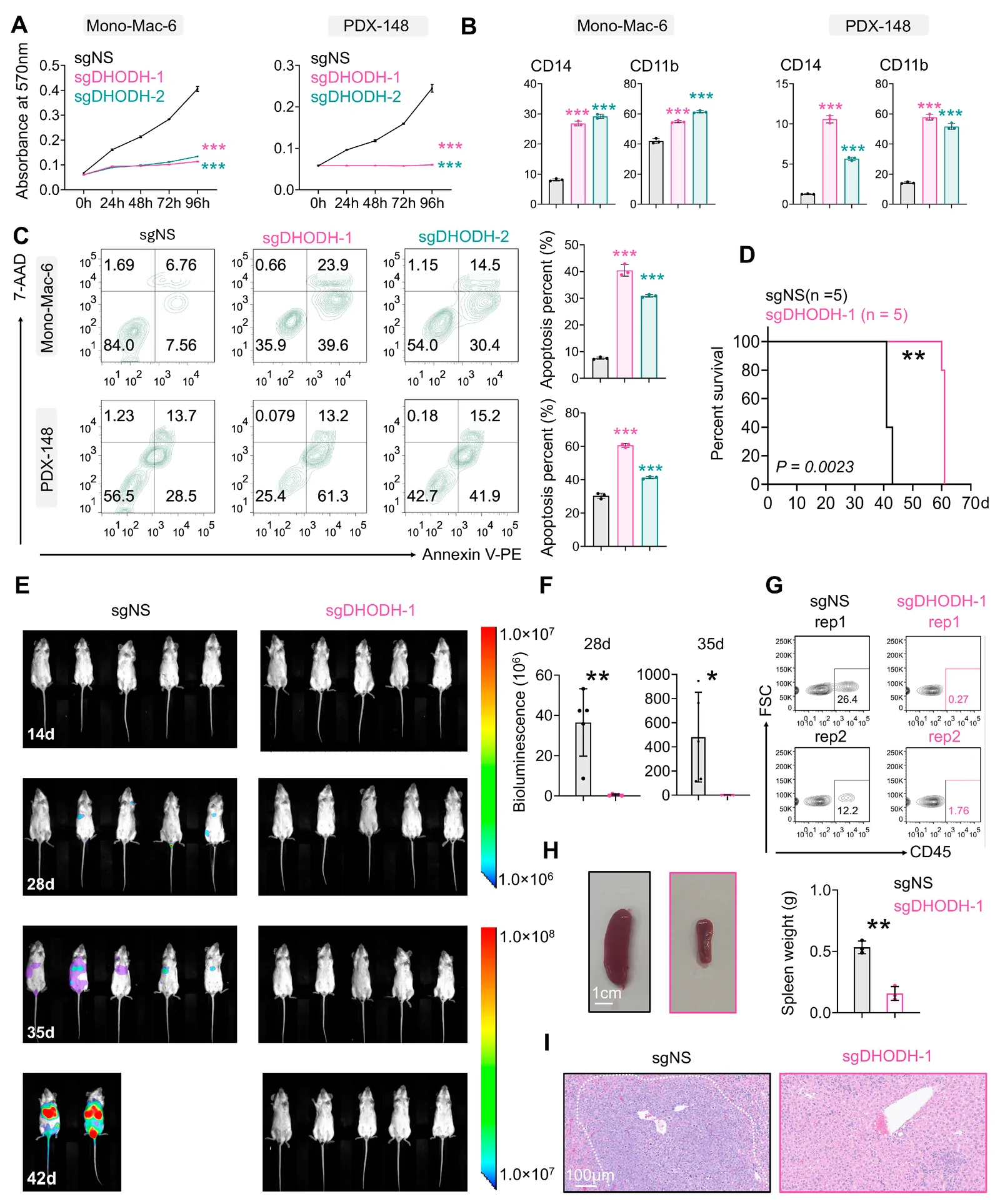
DHODH promotes leukemogenesis. (**A**) The impact of *DHODH* KO on cell growth in the AML Mono-Mac-6 cell line and PDX-148 cells. Two-way ANOVA was utilized. n = 3 replicates. *** *p* < 0.001. (**B**) The impact of *DHODH* KO on cell differentiation in the AML Mono-Mac-6 cell line and PDX-148 cells. One-way ANOVA was utilized. n = 3 replicates. *** *p* < 0.001. (**C**) The impact of *DHODH* KO on cell apoptosis in the AML Mono-Mac-6 cell line and PDX-148 cells. One-way ANOVA was utilized. n = 3 replicates. *** *p* < 0.001. (**D**) Kaplan–Meier survival curves of NSGS mice transplanted with luciferase+ Mono-Mac-6 cells following *DHODH* depletion. The log-rank test was utilized. n = 5 replicates per group. ** *p* < 0.01. (**E**) Representative in vivo bioluminescent images of NSGS mice transplanted with luciferase+ Mono-Mac-6 cells following *DHODH* depletion; 5 × 10^5^ live cells were transplanted for each group. n = 5 replicates per group. (**F**) Quantification of bioluminescence signals on days 28 and 35 after bone marrow transplantation. Two-way ANOVA was utilized. n = 5 replicates per group. * *p* < 0.05; ** *p* < 0.01. (**G**) Representative flow cytometry plots showing AML burden in peripheral blood of Mono-Mac-6 NSGS mice at the disease endpoints. n = 2 replicates per group. (**H**) Representative spleen images and corresponding spleen weights from Mono-Mac-6 NSGS mice at the disease endpoints. Scale bar: 1 cm. An unpaired two-tailed Student’s *t*-test was utilized. n = 3 replicates per group. ** *p* < 0.01. (**I**) H&E staining of liver from Mono-Mac-6 NSGS mice at the disease endpoints. Scale bar: 100 µm.

**Figure 3:**
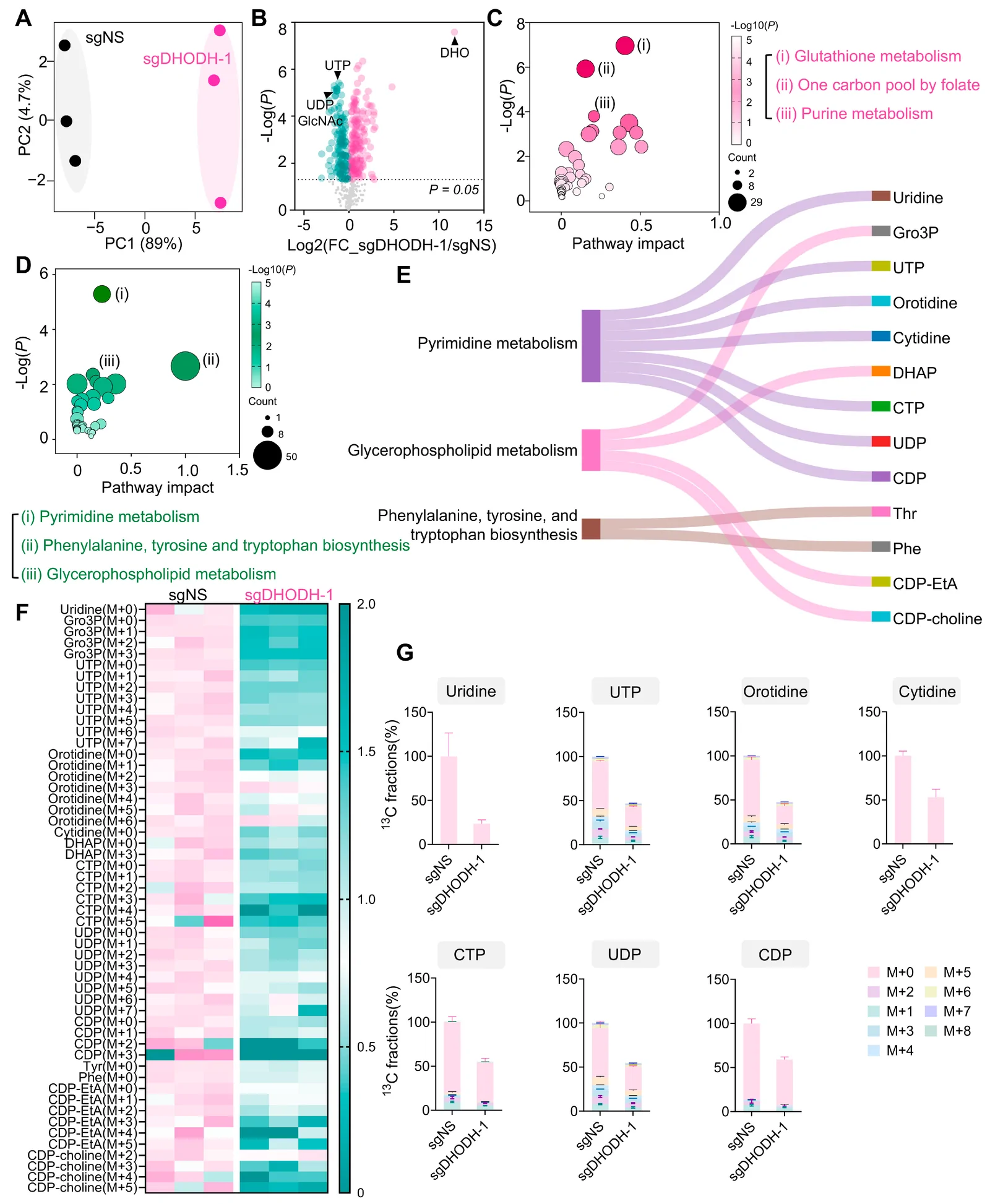
DHODH rewires *de novo* pyrimidine biosynthesis in AML. (**A**) PCA plots of metabolic profiling in Mono-Mac-6 cells following *DHODH* KO (sgDHODH-1). n = 3 replicates per group. (**B**) Volcano plot showing significantly increased (*p* < 0.05) and significantly decreased (*p* > 0.05) metabolites following *DHODH* KO (sgDHODH-1) in Mono-Mac-6 cells. (**C**) Bubble plot showing core-enriched pathways of significantly increased metabolites following *DHODH* KO (sgDHODH-1) in Mono-Mac-6 cells. (**D**) Bubble plot showing core-enriched pathways of significantly decreased metabolites following *DHODH* KO (sgDHODH-1) in Mono-Mac-6 cells. (**E**) Sankey plot of three core-enriched pathways, shown in Figure 3D, and their corresponding 13 metabolites. (**F**) Heatmap showing the relative abundance of the 13 metabolites highlighted in Figure 3E in Mono-Mac-6 cells upon *DHODH* KO (sgDHODH-1). n = 3 replicates per group. (**G**) Metabolic tracing showing isotopolog distributions (M + x, x is the number of ^13^C atoms) of 7 metabolites enriched in the pyrimidine metabolism pathway, shown in Figure 3E, in Mono-Mac-6 cells following *DHODH* KO (sgDHODH-1).

**Figure 4:**
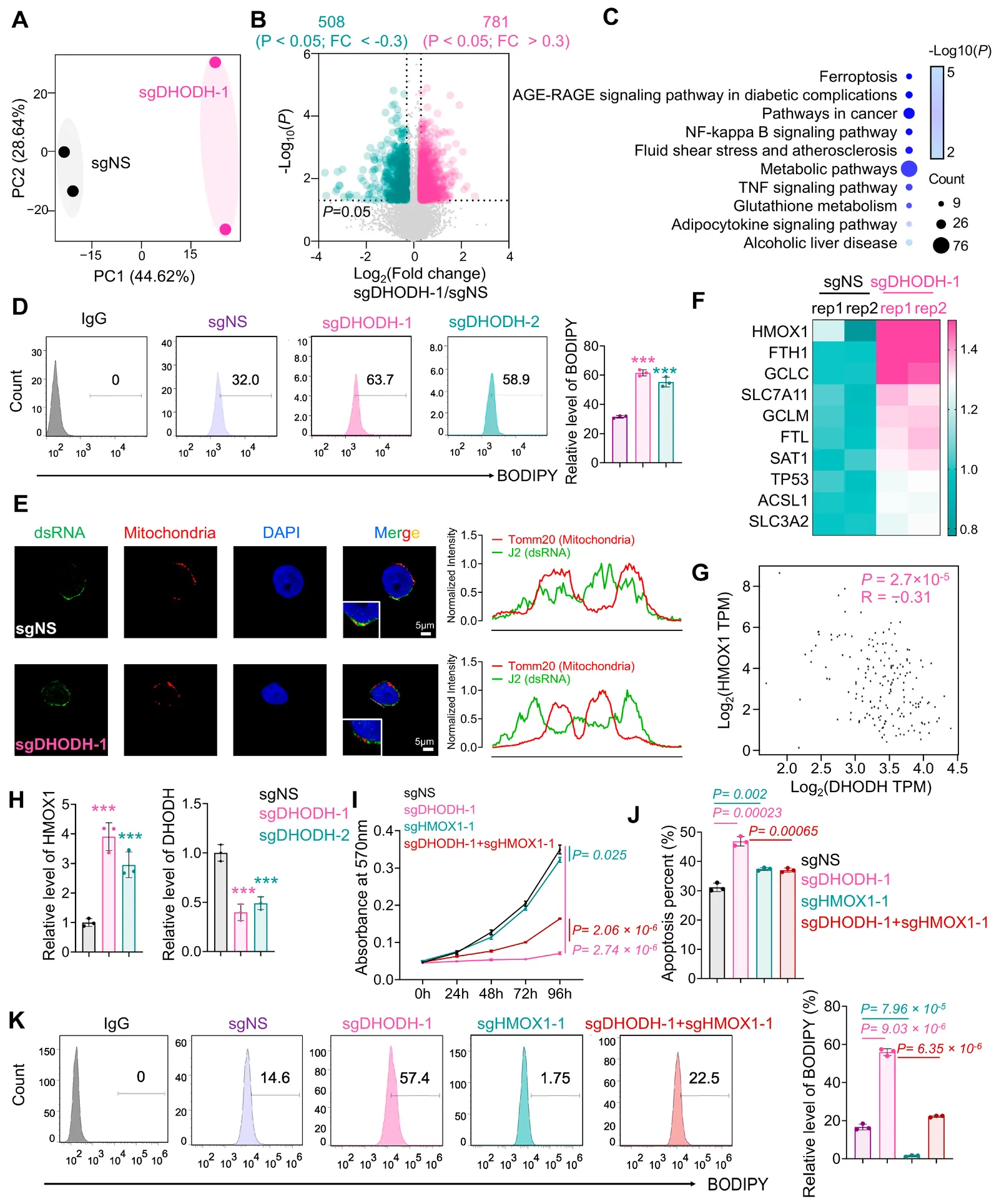
*DHODH* KO induces ferroptosis via upregulating HMOX1 in AML. (**A**) PCA plots of RNA-seq in Mono-Mac-6 cells following *DHODH* KO (sgDHODH-1). n = 2 replicates per group. (**B**) Volcano plot showing significantly increased (*p* < 0.05, Log_2_(FC; sgDHODH/sgNS > 0.3; highlighted in red) and significantly decreased (*p* < 0.05, Log_2_(FC; sgDHODH/sgNS < −0.3; highlighted in green) transcripts following *DHODH* KO (sgDHODH-1) in Mono-Mac-6 cells. (**C**) Pathway analysis showing the top 10 core-enriched pathways of significantly increased transcripts from Figure 4B. (**D**) Flow cytometry analysis showing BODIPY levels in Mono-Mac-6 cells upon *DHODH* KO (sgDHODH-1). Unpaired two-tailed Student’s *t*-tests were used for two-group comparisons. *** *p* < 0.001. n = 3 replicates per group. (**E**) Representative images showing subcellular colocalization (**left**) and corresponding line-scan quantification of fluorescence intensities (**right**) of dsRNA and mitochondria in Mono-Mac-6 cells following *DHODH* KO. Scale bar: 5 µm. Blue: DAPI; green: dsRNA (J2); red: mitochondria (Tomm20). (**F**) Heatmap depicting relative mRNA abundance of 10 genes enriched in the ferroptosis pathway, shown in Figure 4C. (**G**) Correlation of DHODH expression with HMOX1 expression in the TCGA AML cohort. Pearson correlation coefficient (R values). (**H**) RT-qPCR analysis showing *HMOX1* expression level following *DHODH* KO in Mono-Mac-6 cells. n = 3 replicates per group. Unpaired two-tailed Student’s *t*-tests were used for two-group comparisons. *** *p* < 0.001. (**I**) Rescue effect of *HMOX1* KO on cell growth in *DHODH*-deficient Mono-Mac-6 cells. Two-way ANOVA was utilized. n = 3 replicates. (**J**) Rescue effect of *HMOX1* KO on cell apoptosis in *DHODH*-deficient Mono-Mac-6 cells. One-way ANOVA was utilized. n = 3 replicates. (**K**) Rescue effect of *HMOX1* KO on ferroptosis in *DHODH*-deficient Mono-Mac-6 cells. One-way ANOVA was utilized. n = 3 replicates.

**Figure 5:**
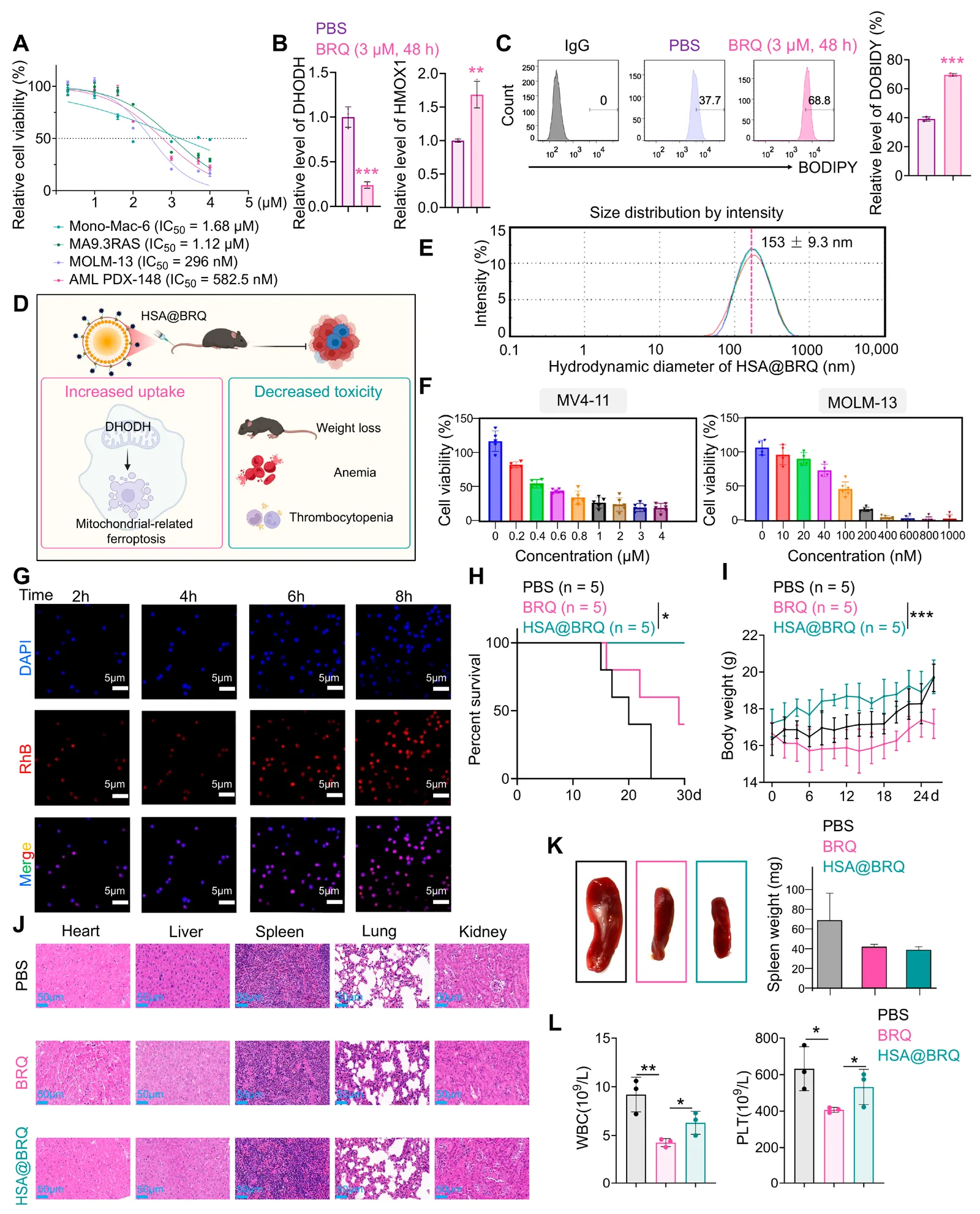
HSA-conjugated BRQ significantly suppressed leukemogenesis while substantially reducing BRQ-induced systemic toxicity. (**A**) IC_50_ values of BRQ for killing AML cell lines and AML PDX cells. The cells were treated with BRQ for 48 h. (**B**) RT-qPCR analysis showing *DHODH* and *HMOX1* expression levels following BRQ treatment in Mono-Mac-6 cells. Unpaired two-tailed Student’s *t*-tests were used for two-group comparisons. ** *p* < 0.01; *** *p* < 0.001. n = 3 replicates per group. (**C**) Flow cytometry analysis showing BODIPY levels in Mono-Mac-6 cells following BRQ treatment. n = 3 replicates per group. *** *p* < 0.001 (**D**) Schematic showing HSA-based BRQ delivery strategy. (**E**) Average diameter of HSA-conjugated BRQ nanoparticles. n = 3 replicates. (**F**) Effect of HSA-conjugated BRQ on killing AML MV4-11 and MOLM-13 cells. n = 3 replicates. (**G**) Representative images showing intracellular delivery efficiency of HSA-conjugated BRQ in MOLM-13 cells. Scale bar: 5 µm. Blue: DAPI; red: RhB-HSA-conjugated BRQ. (**H**) Kaplan–Meier survival curves of the MOLM-13 mouse model following treatment with PBS, BRQ, and HSA-conjugated BRQ. n = 5 replicates per group. Log-rank analysis was utilized. * *p* < 0.05. (**I**) Body weight of mouse model with PBS, BRQ, and HSA-conjugated BRQ. n = 5 replicates per group. Log-rank analysis was utilized. *** *p* < 0.001. (**J**) H&E staining of heart, liver, spleen, lung, and kidney from the mouse model following treatment with PBS, BRQ, and HSA-conjugated BRQ. Scale bar: 50 µm. (**K**) Representative spleen images and corresponding spleen weights from the MOLM-13 mouse model following treatment with PBS, BRQ, and HSA-conjugated BRQ. n = 5 replicates per group. (**L**) The levels of WBC and PLT in the MOLM-13 mouse model following treatment with PBS, BRQ, and HSA-conjugated BRQ. n = 3 replicates per group. Unpaired two-tailed Student’s *t*-tests were used for two-group comparisons. * *p* < 0.05; ** *p* < 0.01.

## Data Availability

Detailed analytical procedures, model performance results, and experimental results supporting the principal findings are provided in Methods and Supplementary Materials. RNA-seq was uploaded to GSE346328.

## Supplementary Materials

The following supporting information can be downloaded at: https://www.mdpi.com/article/10.3390/cancers18193091/s1, Table S1: Metabolic profiling in Mono-Mac-6 cells upon *DHODH* KO; Table S2: RNA-seq in Mono-Mac-6 cells upon *DHODH* KO. Figure S1: The rescue effect of exogenous Uridine on *DHODH* inhibition-induced phenotype in Mono-Mac-6 cells. Figure S2: Variation in the hydrodynamic size of HSA-BRQ nanoparticles with incubation time. Figure S3: Cumulative BRQ release profile in pH 7.4 PBS.
