# Supplementary material for: Pharmacologically Targeting DHODH-Dependent Pyrimidine Metabolism with HSA-Conjugated BRQ Suppresses Leukemogenesis
Source: Cancers (Basel). 2026 Sep 23;18(19):3091. doi: 10.3390/cancers18193091 (PMC13664278; doi:10.3390/cancers18193091)
Supplement: Supplementary file 1 [file cancers-18-03091-s001.zip › Supplementary figures.pdf]

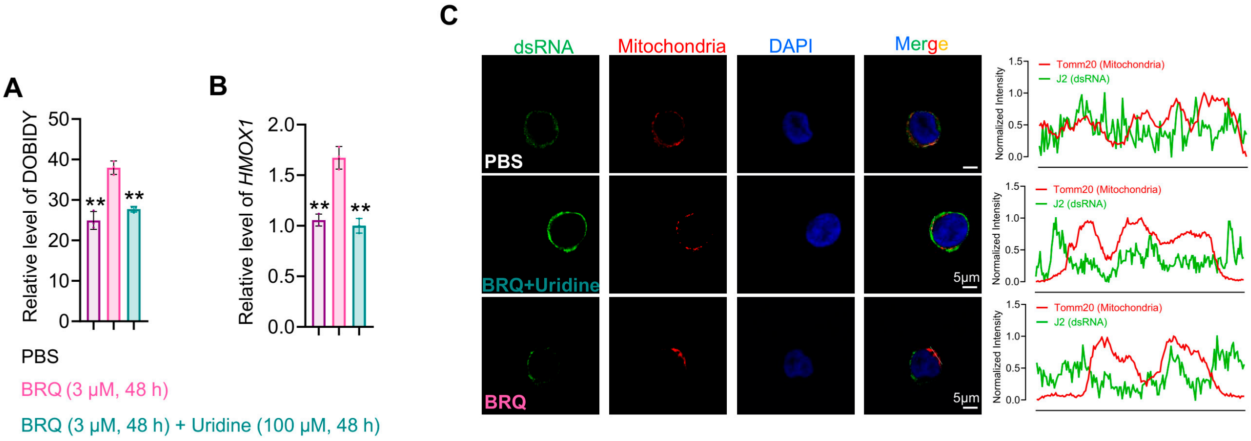

Figure S1. Rescue effect of Uridine in DHODH-inhibited AML cells. (**A**) Rescue effects of exogenous Uridine on ferroptosis in Mono-Mac-6 cells. Two-way Anova analysis was utilized; **p < 0.01. (**B**) Rescue effects of exogenous Uridine on HMOX1 mRNA expression in Mono-Mac-6 cells. Two-way Anova analysis was utilized; **p < 0.01. (C) Rescue effects of exogenous Uridine on mt-dsRNA localization in Mono-Mac-6 AML cells.

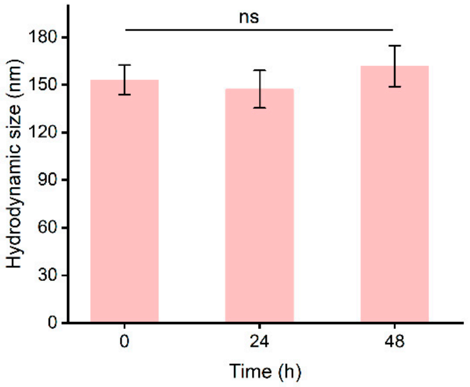

Figure S2. Variation in the hydrodynamic size of HSA-BRQ nanoparticles with incubation time.

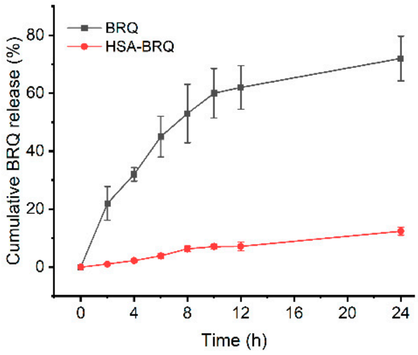

Figure S3. Cumulative BRQ release profile in pH 7.4 PBS.
